# Human induced pluripotent stem cell line banking for the production of rare blood type erythrocytes

**DOI:** 10.1186/s12967-020-02403-y

**Published:** 2020-06-12

**Authors:** Yu Jin Park, Su-Hee Jeon, Hyun-Kyung Kim, Eun Jung Suh, Seung Jun Choi, Sinyoung Kim, Hyun Ok Kim

**Affiliations:** 1 Department of Laboratory Medicine, Yonsei University College of Medicine, 50-1 Yonsei-ro, Seodaemun-gu, Seoul, 03722 Republic of Korea; 2 Department of Laboratory Medicine, Armed Forces Yangju Hospital, Yangju-si, Gyeonggi-do, Korea

**Keywords:** Induced pluripotent stem cell, Rare blood type, Red blood cell differentiation, Stem cell banking

## Abstract

**Background:**

The in vitro production of mature human red blood cells (RBCs) from induced pluripotent stem cells (iPSCs) has been the focus of research to meet the high demand for blood transfusions. However, limitations like high costs and technological requirements restrict the use of RBCs produced by iPSC differentiation to specific circumstances, such as for patients with rare blood types or alloimmunized patients. In this study, we developed a detailed protocol for the generation of iPSC lines derived from peripheral blood of donors with O D-positive blood and rare blood types (D–and Jr(a-)) and subsequent erythroid differentiation.

**Methods:**

Mononuclear cells separated from the peripheral blood of O D-positive and rare blood type donors were cultured to produce and expand erythroid progenitors and reprogrammed into iPSCs. A 31-day serum-free, xeno-free erythroid differentiation protocol was used to generate reticulocytes. The stability of iPSC lines was confirmed with chromosomal analysis and RT-PCR. Morphology and cell counts were determined by microscopy observations and flow cytometry.

**Results:**

Cells from all donors were successfully used to generate iPSC lines, which were differentiated into erythroid precursors without any apparent chromosomal mutations. This differentiation protocol resulted in moderate erythrocyte yield per iPSC.

**Conclusions:**

It has previously only been hypothesized that erythroid differentiation from iPSCs could be used to produce RBCs for transfusion to patients with rare blood types or who have been alloimmunized. Our results demonstrate the feasibility of producing autologous iPSC-differentiated RBCs for clinical transfusions in patients without alternative options.

## Background

Since the 17th century, the transfusion of red blood cells (RBCs) has been a crucial part of modern medicine, enabling the alleviation of symptoms in patients with severe anemia or trauma. Currently, RBCs are only available from donations by healthy volunteers, but insufficient numbers of donors and the potential for transfusion-transmitted infections remain considerable challenges in meeting the demand for blood [1–3]. Despite extensive efforts, it is not always easy to find a suitable blood component, and alternative systems capable of transporting oxygen to the body have been evaluated, including perfluorochemical-based RBC substitutes, hemoglobin-based RBC substitutes, and recombinant hemoglobin [4]. However, these blood substitutes result in insufficient oxygen delivery and an increased likelihood of death [5], and therefore are not promising alternatives [6].

During the past decade, enormous progress has been made in the ex vivo manufacture of human RBCs [7–11]. Hematopoietic stem cells (HSC) obtained from cord blood and bone marrow have been used to produce functional enucleated RBCs [12–16]. There have been numerous advances in the generation of safe erythroid cells and improvements in the efficiency of single hematopoietic cell acquisition by differentiation of CD34 + HSCs using cytokines and small molecules [9, 17–28]. As human HSCs are a limited resource and ethical concerns hinder the use of embryonic HSCs, the large-scale expansion of RBCs for transfusion purposes remains problematic.

Induced pluripotent stem cells (iPSCs) are an essentially infinite source of stem cells owing to their self-renewal ability. Since the first report of these cells in 2006, human iPSCs have been derived from many cell types, including blood cells [29–32]. The original protocol, which involved the integration of transcription factor combinations into the cell genome, has been refined and now makes use of integration-free factors, which make iPSCs therapy for regenerative medicine applications safer [33]. Although the current methods for manipulating iPSCs are extremely inefficient and costly, iPSCs have certain benefits such as immortality, the lack of allogenic immune reactions, and the lack of ethical concerns on destruction of human embryos; therefore, studies of iPSC-derived RBC production are ongoing. An ideal strategy is to generate autologous RBCs using iPSCs, but the process is not feasible in clinical settings owing to the long time required for supplying the manufactured blood for transfusion and high cost of production.

Irrespective of the number of blood donors, maintaining an all-inclusive supply of transfusable blood for the broad population is challenging, particularly owing to alloimmunized individuals or those with rare blood types. Thirty blood group systems and over 300 RBC alloantigens have been identified, but most alloantibodies belong to the Rh, Kell, Duffy, Kidd, and MNS blood group systems [34]. The development of “universal” RBCs applicable to the entire population has been slow [35]. A study by the French National Registry of People with a Rare Blood Phenotype/Genotype suggested that 15 iPSC lines are sufficient to manage nearly all alloimmunized patients [36].

Blood banks have difficulty in finding suitable blood components for patients with rare blood types such as Jr(a-) and Dphenotypes. Jr^a^ is an extremely high-incidence antigen seen in all populations. The incidence of Jr(a-) phenotype is only 0.03 to 0.12 percent even in Japanese population, which has been noted to have the highest frequency of Jr(a-) individuals worldwide  [37]. D–phenotypes are characterized by red blood cells not expressing RHCE protein at their plasma membrane, which leads to absence of C, c, E, and e antigens. Although this phenotype can be found in various populations, it is very rare with frequencies estimated at 0.001 percent in Japanese population [38]. Therefore, the use of iPSCs prepared from patients with rare blood types to obtain RBCs has been considered [39–42], but empirical evidence for the effectiveness and safety of this approach are lacking.

In this study, we constructed a registry of iPSC lines from peripheral blood samples collected from donors with the O D-positive and rare blood group phenotypes. We devised a detailed protocol for generating iPSCs from peripheral blood mononuclear cells (PB-MNCs) and differentiating them into functional RBCs.

## Materials and methods

### Study design and cell sources

After receiving consent from patients and donors, blood was collected from five O D-positive donors and from two patients with rare blood types, D–and Jr(a-). RBC antigenic phenotypes were determined by using monoclonal RBC antibody reagents (Ortho-Clinical Diagnostics, Raritan, NJ, USA). The H9 human embryonic stem cell line (WiCell, Madison, WI, USA) was used as a control. This study was approved by the Institutional Review Board of Yonsei University Severance Hospital, Seoul, Korea (IRB No. 4-2016-1158).

### Generation of induced pluripotent cell lines

#### Isolation of pb-mncs from whole blood

Peripheral blood (10–15 mL) was drawn into a tube containing sodium heparin anticoagulant (BD Biosciences, Oxford, UK). PB-MNCs were purified using either Ficoll–Paque Premium (GE Healthcare, Uppsala, Sweden) or Lymphoprep (Stem Cell Technologies, Oslo, Norway). Viable PB-MNCs were counted by the trypan blue exclusion method (Trypan Blue Stain 0.4%; Gibco, Life Technologies, Carlsbad, CA, USA) [43].

#### Expansion of erythroid progenitors

To stimulate the growth of erythroid precursors, viable PB-MNCs were resuspended at a density of 1 × 10^6^ cells/mL in erythroid expansion medium, composed of basal medium and erythroid cytokines. The reagents used for cell cultures have been listed in Table 1. The basal medium was prepared by adding 150 μg/mL transferrin (Sigma-Aldrich, Gillingham, UK), 50 μg/mL insulin (Sigma-Aldrich), 90 ng/mL ferrous nitrate (Sigma-Aldrich), 160 μM monothioglycerol (Sigma-Aldrich), and 1% penicillin–streptomycin (Gibco) in Stemline II medium (Sigma-Aldrich). The erythroid expansion medium was prepared by adding 1 μM hydrocortisone (Sigma-Aldrich), 100 μg/mL stem cell factor (Sigma-Aldrich), 6 IU/mL erythropoietin (StemCell Technologies, Vancouver, Canada), and 10 μg/mL interleukin 3 (Peprotech EC Ltd., London, UK) to the basal medium. If there were excess PB-MNCs, the surplus cells were frozen with either Cryostar (BioLife Solutions, Bothell, WA, USA) or mFreSR (StemCell Technologies).

**Table 1:** List of reagents used for cell culture

Materials	Abbreviations	Company	Catalogue#
Bone morphogenetic proteins 4	BMP4	Peprotech	120-05
Vascular endothelial growth factor	VEGF	Peprotech	100-20
Wnt3A		Peprotech	315-20
Activin A		Peprotech	120-14E
GSK-3β Inhibitor VIII		Calbiochem	361549
Fibroblast growth factor alpha	FGFa	Peprotech	100-17A
β-Estradiol		Sigma-Aldrich	E2257
Insulin-like growth factor 2	IGF2	Peprotech	100-12
Thrombopoietin	TPO	Peprotech	300-18
Heparin		StemCell Technologies	07980
3-Isobutyl-1-methylxanthine	IBMX	Sigma-Aldrich	I5879
StemRegenin 1	SR1	Cellagen Technology	C7710
Hydrocortisone	HC	Sigma-Aldrich	H0888
Stem cell factor	SCF	Peprotech	300-07
Interleukin 3	IL-3	Peprotech	200-03
Erythropoietin	EPO	Stem Cell	02625
Poloxamer 188	P188	Sigma-Aldrich	P5556
Transferrin	TF	Sigma-Aldrich	T8158
Insulin		Sigma-Aldrich	I3536
Ferric nitrate	FN	Sigma-Aldrich	F8508
Monothioglycerol	MTG	Sigma-Aldrich	M6145
Penicillin–Streptomycin	P-S	Gibco	15140122
Y-27632		StemCell Technologies	72392
Stemline II hematopoietic stem cell expansion medium		Sigma-Aldrich	S0192
mTeSR1 basal medium		StemCell technologies	85851
mTeSR1 5 × Supplement		StemCell technologies	85852
AggreWell EB formation medium		StemCell technologies	05893

MNCs (1 × 10^7^) were suspended in 10 mL of erythroid expansion medium in a 25T (Nunc EasYFlask Cell Culture Flask, Cat# 156367; Thermo Scientific, Waltham, MA, USA) flask and cultured for 3 days in a 5% CO_2_ incubator at 37 °C. After 3 days, both non-adherent and adherent cells were recovered, and the cells were resuspended at a density of 1 × 10^6^ cells/mL in fresh erythroid expansion medium. From day 7, morphological analyses were performed daily until the population of erythroid progenitor cells accounted for more than 80% of the total PB-MNCs. When the population of erythroid progenitor cells reached 80% or higher, the cells were ready to be transfected. The erythroid enrichment step can be prolonged to obtain more erythroid progenitor cells in fresh erythroid expansion medium.

#### Reprogramming of expanded erythroid progenitors

Before transfection, each well in a 6-well multidish (Nunc Cell-Culture Treated Multidish, Cat#140675; Thermo Scientific) was coated with a mixture of 14.5 μL of Matrigel Matrix (Corning, Kennebunk, ME, USA) and 985.5 μL of DMEM/F12 (1 ×) (Gibco) for 1 h at 25 °C. A total of 1 × 10^6^ culture-expanded erythroid cells were centrifuged (400×*g*, 5 min) and resuspended in 104 μL of medium (18 μL of supplement 1, 82 μL of nucleofector solution (both from the P3 Primary Cell 4D-Nucleofactor Kit, Lonza Amaxa), 2 μL of Epi5 Episomal Reprogramming Vectors, and 2 μL of Epi5 p53 and EBNA vectors (both from the Epi5 Episomal iPSC Reprogramming Kit; Life Technologies, Frederick, MD, USA) at 25 °C for 10 min. Prepared cells were transferred to a 100-μL Nucleocuvette Vessel (Lonza, Koln, Germany) and loaded on the 4D-Nucleofector System (Lonza). Electrotransfection under the ‘CD34 cell, human cell type’ program as per the manufacturer’s instructions (https://bioscience.lonza.com/lonza_bs/US/en/download/product/asset/30292). After removing the cuvette, the processed cells were transferred to 6 mL of erythroid expansion medium, mixed well, and plated at a density of 3.3 × 10^5^ cells per well (i.e., 2 mL of the cell suspension) on a Matrigel pre-coated 6-well plate.

Each well was supplemented with 1 mL of erythroid expansion medium on post-transfection day (PT-D) 2, and with 1 mL of ReproTeSR Basal Medium (Stem Cell Technologies) on PT-D3 and PT-D5. From PT-D7, complete medium changes were performed with 2 mL of ReproTeSR daily, and the cultures were close observed until colonies with an iPSC-like appearance were observed. Typically, iPSC-like colonies appeared after PT-D14. The colonies were manually picked based on their morphology between PT-D14 and PT-D24 under a polarizing microscope, and each colony was passaged as individual iPSC lines thereafter.

#### Maintenance of induced pluripotent stem cells

Human iPSC cultures were maintained on plates coated with 40 μL of Vitronectin XF (Stem Cell Technologies) and 1 mL of CellAdhere Dilution Buffer (Stem Cell Technologies) for 1 h in 2 mL of mTESR1 medium (mTeSR1 5 × Supplement 1:4 mTeSR1 Basal medium; Stem Cell Technologies). All cells were cultured at 37 °C in a humidified atmosphere containing 5% CO_2_ and were cultured daily with mTESR1 media until reaching 80–90% confluence. The cells were typically ready for passage within 5 to 7 days. For the newly reprogrammed iPSCs (i.e., up to passage 3), colonies were mechanically passaged using a drawn-out glass Pasteur pipette to dissociate individual colonies. This method is used for passaging only desired undifferentiated iPSC colonies, and not unwanted differentiated colonies. From passage 3, iPSCs were enzymatically passaged using ReLeSR (Stem Cell Technologies). Medium was changed daily, and cells were subcultured once every 5 to 7 days.

### Differentiation into erythrocytes

#### Generation of embryoid bodies (EB) (Fig. 1)

On differentiation day (DD) 0, EBs were formed on AggreWell 400 plates (Stem Cell Technologies) according to the manufacturer’s instructions (https://cdn.stemcell.com/media/files/manual/MA29146-Reproducible_Uniform_Embryoid_Bodies_Using_AggreWell_Plates.pdf). AggreWell plates were pre-treated with 2 mL of AggreWell Rinsing Solution to remove any bubbles lodged within the wells (centrifugation at 2000×*g*, 5 min). Single cells were derived from the monolayer culture of iPSCs by enzymatic treatment with Easy Gentle Cell Dissociation Reagent (Stem Cell). Approximately 2 × 10^6^ single cells were seeded into each well of the AggreWell plate with 5 mL of AggreWell EB Formation Medium (Stem Cell Technologies) containing 10 μM Y-27632 (Stem Cell Technologies). Cell aggregation was achieved by centrifugation at 100 × g for 3 min.

**Fig. 1:**
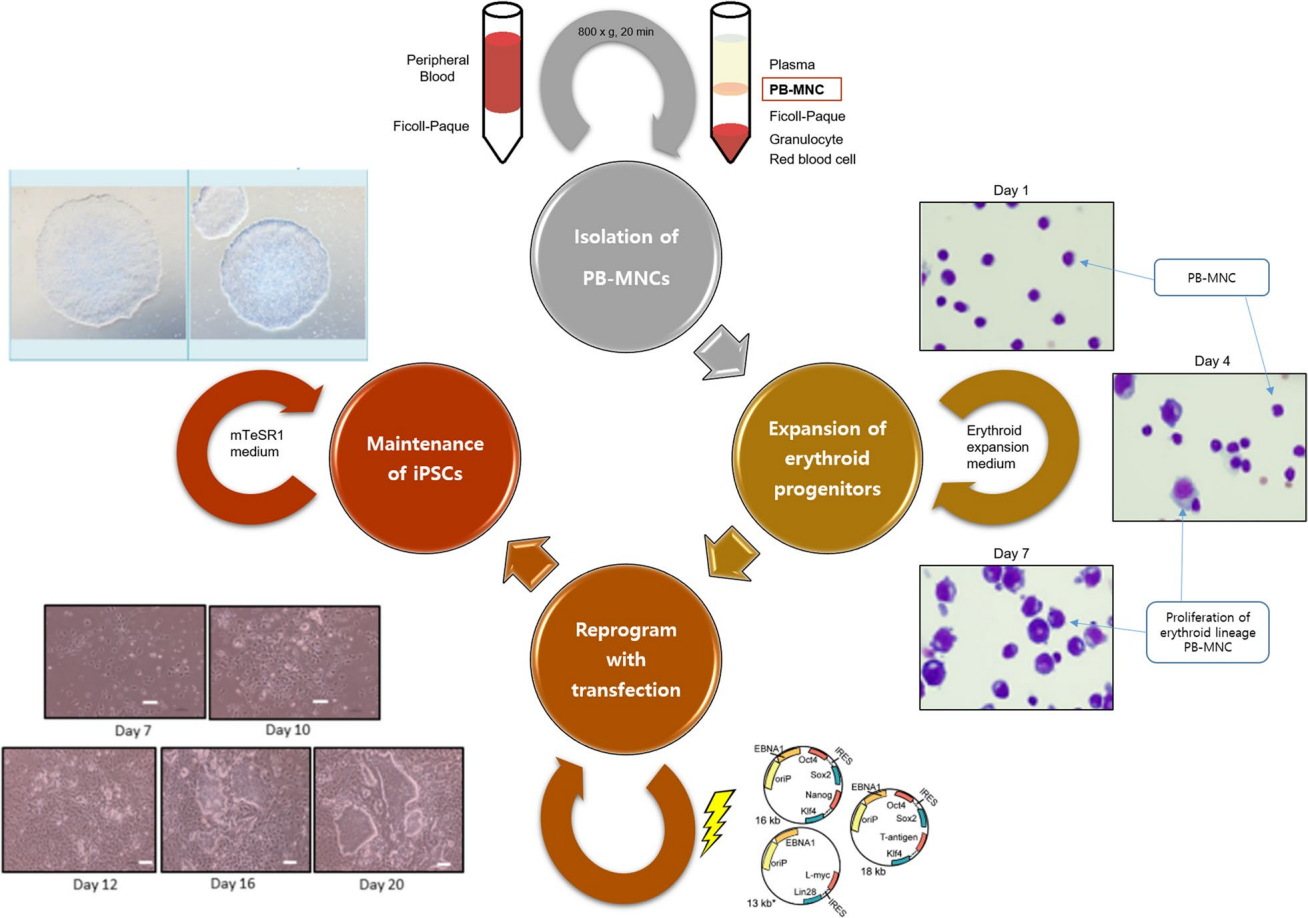
The scheme of the production of human induced pluripotent stem cells from peripheral blood. After isolating the PB-MNCs using Ficoll-Paque, cells are enriched in erythroid expansion medium for 7 days to stimulate the growth of erythroid precursors. When erythroid progenitor cells reach 80% of the total PB-MNCs, cells are transfected with reprogramming vectors (Oct4, Sox2, Lin28, Klf4, and L-Myc)

On DD1, EBs from each well of the AggreWell plate were transferred to a 6-well multi-dish with Stemline II medium containing 5 ng/mL bone morphogenic protein 4 (BMP4), 5 ng/mL vascular endothelial growth factor (VEGF), 2.5 ng/mL Activin A, 5 ng/mL Wnt3A, and 0.5 μl/ml GSK3β inhibitor VIII. Cells were plated at 5 × 10^5^ cells per well.

On DD3, a new set of cytokines was added to the existing culture volume along with 0.5 mL of Stemline II medium per well. Cytokines were added at a 6× concentration to supplement the entire volume such that the final concentrations in the well (assumed volume, 3 mL) were 20 ng/mL BMP4, 30 ng/mL VEGF, 10 ng/mL Wnt3A, 5 ng/mL Activin A, 2 mM GSK3β inhibitor VIII, 10 ng/mL acidic fibroblast growth factor (FGFa), 20 ng/mL stem cell factor (SCF), and 0.4 ng/mL β-estradiol.

#### Differentiation towards hematopoietic stem cell lineage (Fig. 2)

On DD4, EBs were harvested, washed in DPBS, and dissociated using TrypLESelect × 10 (Gibco, Thermo Scientific) for 10 min at 37 °C. The cells were resuspended in fresh Stemline II medium and plated at 2 × 10^5^ cells per well of a standard six-well tissue culture plate with the following factors: 20 ng/mL BMP4, 30 ng/mL VEGF, 10 ng/mL FGFa, 30 ng/mL SCF, 10 ng/mL insulin-like growth factor 2, 10 ng/mL thrombopoietin, 5 μg/mL heparin, 50 mM isobutylmethyl xanthine, and 0.4 ng/mL β-estradiol.

**Fig. 2:**
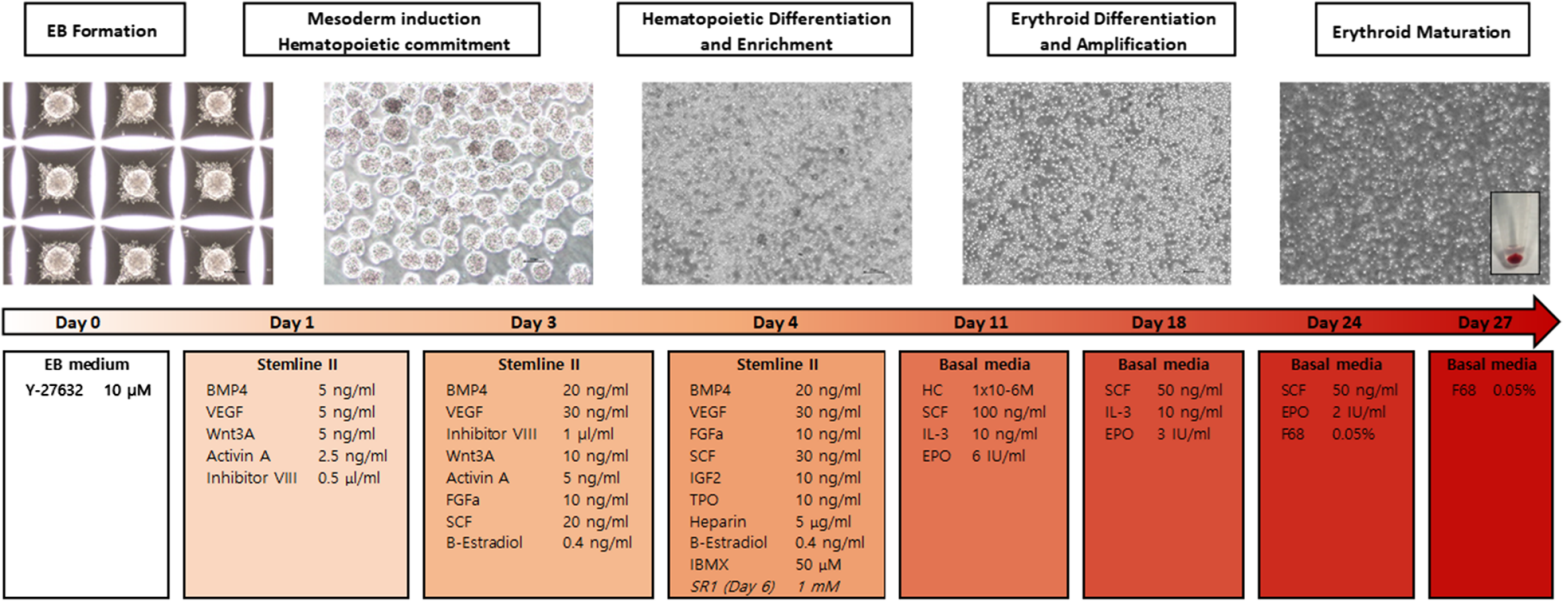
Diagram representing the feeder-free and serum-free erythroid differentiation of iPSCs. Abbreviations: BMP, bone morphogenic protein; EB, embryoid body; EPO, erythropoietin; FGF, fibroblast growth factor; HC, hydrocortisone; iPSC, induced pluripotent stem cells; IBMX, isobutylmethyl xanthine; IGF, insulin-like growth factor; IL, interleukin; SCF, stem cell factor; TPO, thrombopoietin; VEGF, vascular endothelial growth factor

On DD6, the differentiation factors from DD4 were refreshed by adding 0.5 mL Stemline II medium per well with a 6× concentration of cytokines and StemRegenin (1 mM) was added. On DD8, cells were subjected to a complete medium change with DD4 factors. If the cell count was greater than 5 × 10^5^ cells/mL, the culture was split, and the density was reset to 2 × 10^5^ cells/mL to support proliferation. On DD9, half of DD4 factors were added in 0.5 mL of Stemline II medium per well.

#### Differentiation towards erythroid precursors and maturation

On DD11, the cells were replated in erythroid liquid culture conditions. The cells were plated at a density of 3 × 10^6^ cells per well in 3 mL of basal medium with the following cytokines: 1 μM HC, 100 ng/mL SCF, 10 ng/mL IL-3, and 6 IU/mL EPO. On DD14, the cells were subjected to a complete medium change with DD11 factors. On DD18, the cells were replated at a density of 3 × 10^6^ cells per well in 3 mL of basal medium with the following cytokines: 50 ng/mL SCF, 10 ng/mL IL-3, and 3 IU/mL EPO. On DD21, medium was completely replaced with DD18 factors. On DD24, the cells were replated at a density of 3 × 10^6^ cells per well in 3 mL of basal medium with the following cytokines: 50 ng/mL SCF, 2 IU/mL EPO, and 0.05% poloxamer 188. On DD27, the cells were replated at a density of 3 × 10^6^ cells per well in 3 mL of basal medium with 0.05% P188.

### Detection of transfected reprogramming factor genes

Total RNA from iPSCs was isolated using the RNeasy Plus Mini Kit (Qiagen, Hilden, Germany). Two micrograms of total RNA were used for reverse transcription reaction using the SuperScript III First Strand (Invitrogen, Thermo Scientific) according to the manufacturer’s instructions. Quantitative real-time RT-PCR (qRT-PCR) was performed using qPCR TaqMan probes (Applied Biosystems) and Step One Plus (Applied Biosystems, Foster City, CA, USA). All experiments were performed in duplicate, and a non-template control (lacking the cDNA template) was included in each assay. Gene expression levels were normalized relative to levels of endogenous glyceraldehyde 3-phosphate dehydrogenase (*GAPDH*) and relative expression was calculated using the ΔΔC_T_ method  [44].

The following qPCR TaqMan probes (Applied Biosystems) were used: POU5F1(OCT4) Hs04260367_gH_FAM; NANOG Hs02387400_g1_FAM; SOX2 Hs01053049_s1_FAM; KLF4 Hs00358836_m1_FAM; c-MYC Hs00153408_m1_FAM; GAPDH Hs02758991_g1_VIC.

### Immunocytochemistry assay

Reprogrammed cells were fixed in 4% paraformaldehyde (Tech & Innovation, Gangwon-do, Korea) for 20 min at room temperature, washed twice with DPBS, and permeabilized with 0.2% Triton X-100 (Sigma-Aldrich) for 15 min at room temperature. The cells were blocked for 1 h with 5% donkey serum in DPBS. Samples were incubated at room temperature for 1 h with primary antibodies against stage-specific embryonic antigen 4 (SSEA4), octamer-binding transcription factor 4 (OCT4), sex-determining region Y-box 2 (SOX2), TRA-1-60, and NANOG homeobox (NANOG) (all from Human Embryonic Stem Cell Marker Panel, Abcam, Cambridge, UK) (Table 2). Secondary antibodies, either Alexa Fluor 594 anti-rabbit or Alexa Fluor 488 anti-mouse antibodies (Life Technologies, Eugene, OR, USA), were incubated at room temperature for 1 h. The nuclei were stained with 4′6-diamidino-2-phenylindole (VYSIS, Downers Grove, IL, USA). Cells were visualized under a fluorescence microscope (CKX53; Olympus, Tokyo, Japan) with an Olympus U-RFL-T fluorescence lamp. Image analysis and colocalization studies were performed using Ocular Image Acquisition Software (OCULAR, version 2.0.1.496; Digital Optics Limited, Auckland, New Zealand).

**Table 2:** List of antibodies used for immunocytochemistry or flow cytometry

Antibodies used for immunocytochemistry		
Marker type	Antibody	Company, Cat#
Embryonic stem cell markers	Rabbit anti-human OCT4	Abcam, Cat# ab109884
	Rabbit anti-human SOX2	
	Rabbit anti-human NANOG	
	Mouse anti-human TRA-1-60	
	Mouse anti-human SSEA4	
Secondary antibodies	Alexa Fluor 594 donkey anti-rabbit IgG	Life Technologies, Cat# A-21207
	Alexa Fluor 488 goat anti-mouse IgG	Life Technologies, Cat# A-11001

Antibodies used for flow cytometry		
Embryonic stem cell markers	PE mouse anti-human TRA-1-60	BD Biosciences, Cat# 560193
	FITC mouse anti-human SSEA4	BioLegend, Cat# 330410
Differentiation markers	FITC mouse anti-human CD34	BD Biosciences, Cat# 555821
	APC mouse anti-human CD43	BD Biosciences, Cat# 560198
	PE mouse anti-human CD235a	BD Biosciences, Cat# 555570
	APC mouse anti-human CD71	BD Biosciences, Cat# 551374
Isotype control	FITC mouse IgG	BioLegend, Cat# 401306
	PE mouse IgM	BD Biosciences, Cat# 555584
	APC mouse IgG	BD Biosciences, Cat# 555751

APC, allophycocyanin; Cat, catalogue; FITC, fluorescein isothiocyanate; PE, phycoerythrin

### Flow cytometric analysis

At DD0, human iPSCs were analyzed by flow cytometry for investigating the expression of the pluripotency markers SSEA4 and TRA-1-60 (BD Biosciences). iPSCs were dissociated using Gentle Cell Dissociation Reagent (Gibco, Thermo Scientific) and aliquots of 1 × 10^5^ cells/200 μL (0.5 M EDTA, pH 8.0, 1:90 DPBS) were prepared. Conjugated antibodies (10 μL/10^5^ cells) were added to the cells and incubated on ice for 30 min in the dark. Unbound antibodies were removed by washing the cells with 900 μL of DPBS, centrifugation at 160×*g* for 5 min, and decanting the supernatant. Cells were resuspended in 400 μL of 4% paraformaldehyde (Tech & Innovation) for preservation up to 3 days.

At DD4, 11, 18, and 24, cells were analyzed by flow cytometry to evaluate their hematopoietic and erythroid characteristics. TrypleSelect × 10 (Gibco, Thermo Scientific) was used to dissociate the cells, if they were not evenly dissociated. Preparation procedures were identical to those used for DD0.

All antibodies used for flow cytometry have been listed in Table 2. The BD FACSVerse Flow Cytometer (BD Biosciences) and FlowJo (version 10.2, FlowJo, LLC, Ashland, OR, USA) were used for the analysis. Nonspecific immunoglobulin isotype controls of the corresponding class served as negative controls. Compensation beads were used to modify compensation matrixes.

### Analysis of chromosomal abnormalities

The cells were fixed and examined by a standard G-banding chromosome analysis [45]. The analysis was performed by GenDix, Seoul, Korea. For each cell line, 20 metaphase cells were analyzed.

### Morphological analysis

Cells (1 × 10^5^ cells per slide) were immobilized onto a glass microscope slide using a cytocentrifuge (Cytospin 4, Thermo Scientific; 800 rpm, 3 min) and stained with Wright-Giemsa dye (Sigma-Aldrich) for observation.

## Results

### Establishment of iPSCs generated from PB-MNCs

**Fig. 3:**
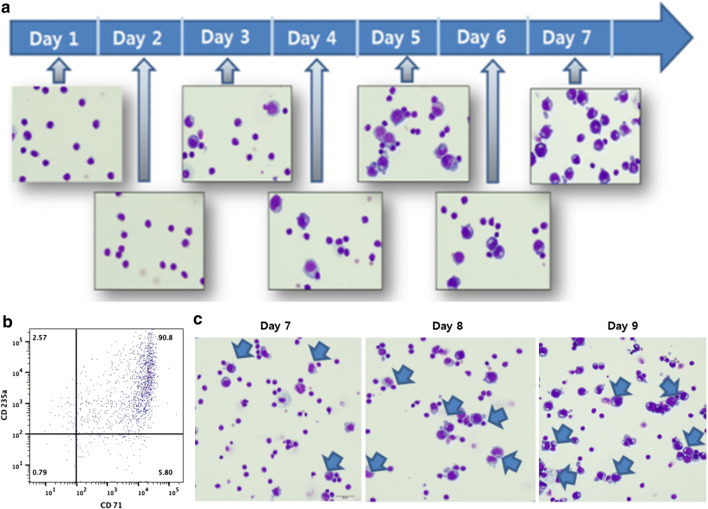
Counting erthyroblast cells to determine the date for transfection: **a** separated PB-MNCs were enriched with cytokines adequate for promoting erythroid progenitors. Typically, erythroblast population exceeded 80% on expansion day 7. **b** flow cytometry analysis of 7-days enriched erythroid progenitors presenting CD235a and CD71 antigens. **c** On erythroblast expansion day 7, if the observed erythroblast population (blue arrow) was less than 80%, transfection was performed after extending the expansion step for 2–3 days in the same conditions

The production of hiPS cell lines from peripheral blood samples involved the following three steps: erythroblast enrichment, electrotransfection, and iPSC initiation. In the erythroblast enrichment step, the cells were transfected when the erythroblast population exceeded 80% (Fig. 3). Typically, cells were ready for transfection on day 7 of the enrichment step as the erythroblast population presenting both CD235a and CD71 antigens usually exceeded 80% by day 7, but if the cells were not ready the enrichment step was prolonged for couple more days. When the erythroblast percentage was between 40% and 50%, the enrichment step was prolonged for 2 to 3 days before transfection.

After transfection, iPSC colony isolation took 7–21 days (mean, 16 days), and individual variation was observed in colony formation efficiency with a yield of 4–10 colonies per 1 × 10^6^ MNCs. The feeder-free transfer method was used for passaging established cell lines. The reprogramming efficiency was quite low (0.001%), but all cultures resulted in the formation of some iPSC colonies.

### Characterization of the stemness of iPSCs generated using episomal vectors

**Fig. 4:**
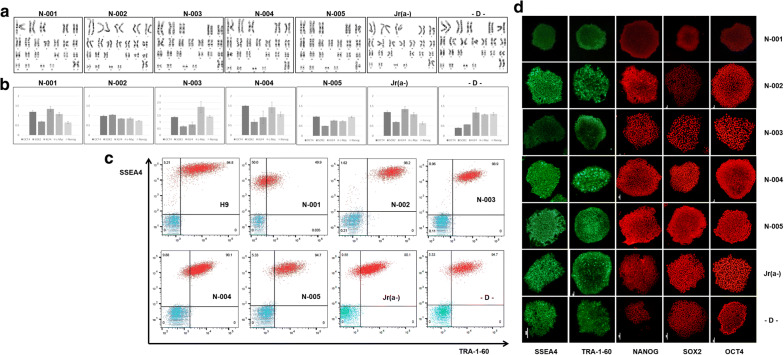
Stemness characterization of iPSCs generated from normal donor (N-001 ~ N-005) and patients with rare blood type (Jr(a-), D–): **a** chromosomal analysis showed normal karyotypes for all cell lines, **b** quantitative reverse transcription polymerase chain reaction revealed successful transfection of reprogramming factor genes, **c** flow cytometric analysis and **d** immunocytochemistry assay indicated that each cell line expressed embryonic stem cell markers (SSEA4, TRA-1-60, NANOG, SOX4, and OCT4)

The stemness of iPSCs was verified using iPSC colonies from passages 8–10. Chromosomal analyses, qRT-PCR, flow cytometry analysis, and immunocytochemical staining of iPSCs were performed for 5 O D-positive subjects and 2 subjects with rare blood (Fig. 4). We established that iPSCs generated from rare blood types using our protocol behave similarly in culture and colony morphologies to those of H9 or O D-positive controls. A chromosomal analysis of all peripheral blood iPSC colonies showed a normal karyotype. Quantitative RT-PCR showed expression of transfected reprogramming factor genes. By flow cytometry analysis, single cells were shown to express pluripotency markers TRA-1-60 and SSEA4. Immunocytochemistry assay revealed that iPSC clones retained the typical characteristics of pluripotent stem cells, including the expression of embryonic stem cell markers (e.g., OCT4, SOX2, NANOG, TRA-1-60, and SSEA4). These data demonstrated the pluripotency of the iPSCs.

### Differentiation of banked iPSCs into erythroid lineage cells

**Fig. 5:**
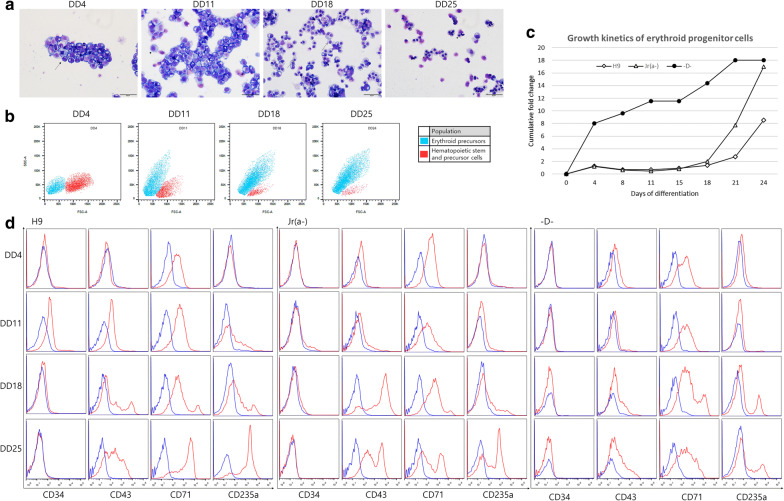
Characteristics of erythroid lineage cells differentiated from donor iPSCs: **a** photographs of Wright-Giemsa staining of cytospin preparations at different stages of the differentiation protocols at days 3, 10, 17, and 24 for Jr(a-) cell line. **b** shift in population of cultured Jr(a-) cells from hematopoietic stem and precursor cells to erythroid precursors. **c** growth kinetics of H9, Jr(a-), and D– cell lines measured as cumulative fold change. **d** chronological shift of CD markers of each cell line (blue) and isotype control (red) as assessed by flow cytometry

Erythroid cell morphology developed over time during the differentiation cultures (Fig. 5a). A shift from a relatively large proportion of hematopoietic precursor cells (HPCs) to differentiated erythroid precursors were observed between DD4 and DD24; during this time, the population shift was evident by flow cytometry analysis (Fig. 5b). The cumulative change in cell number from iPSCs to differentiated cells after 24 days were between 8 and 18-folds (Fig. 5c). Jr(a-) and H9 cell lines showed similar amplification curves until DD15, and then Jr(a-) showed accelerated proliferation reaching up to 17-folds by DD24, whereas H9 stopped at nine-folds. In contrast, D– cell line showed accelerated growth in the beginning of the differentiation protocol, and the final cumulative folds for rare blood groups were similar on DD24, which were higher than that of the H9 cell line.

Using flow cytometry, the markers of HSCs (CD34 and CD43), early erythroid progenitors (CD71), and mature erythroid cells (CD235a) were detected (Fig. 5d). Antigen presentation for each cell lines varied, but some common characteristics could be found. CD34 + CD43 + HSC population was observed in H9 cell lines on DD11, but CD34 antigen was not observed in any of the rare blood type cell lines. In all cell lines, early HPCs expressing CD43 were detected from DD11 onwards [46]. Most HSCs were differentiated into HPCs, whereas HPCs were observed throughout the duration of the differentiation protocol, with a decrease in CD34 and high frequency of CD43. CD71, which is selectively expressed at high levels not only in erythroid precursors but also in all proliferating cells [47], was strongly expressed in all erythroid precursors and even in HSCs. The mature erythroid cell marker, CD235a was prominently observed after DD18 and peaked on DD24, which were more readily observed in H9 and Jr(a-) cell lines. Generally, the order of antigenic profile for the differentiated cells were CD71, followed by CD43 with or without presence of CD34, and finally CD235a + CD71 high population.

## Discussion

Extensive studies have evaluated the differentiation of iPSCs into erythrocytes as a possible strategy to meet the demand for blood components [7, 8, 11]. Various protocols have been developed all of which follow the same basic steps: iPSC generation by incorporating pluripotent genetic factors, self-renewal and expansion of the iPSCs, and differentiation into erythroid lineage cells, which includes commitment, expansion, and maturation [27, 31, 48–50]. Many studies have focused on specific parts of the protocol, but relatively few studies have evaluated the entire process, starting from iPSC generation to erythroid differentiation.

Finding suitable starting population for growing RBCs has been a challenge. The main source materials to produce RBCs in vitro are hematopoietic stem/progenitor cells, embryonic stem cells, and iPSCs [51]. The major advantage of iPSCs over other source materials is that they can be produced from any type of cells, both immature and mature cells without causing ethical concerns arisen by using human embryos [52]. Our experiment used PB-MNCs as the starting population for iPSCs because reprogrammed iPSCs may retain epigenetic memory inherited from the parental cells. Therefore, using hematopoietic cells were preferable to other sources such as fibroblasts.

Assuming that efficient in vitro large-scale cultured RBC production will be possible in the near future, the banking of iPSC clones may be a key strategy for providing iPSC-derived RBCs. Based on this approach, our laboratory has started to build a registry of iPSC clones from many O D-positive and O D-negative donors. By confirming that the antigen profiles of donor RBCs match those of differentiated RBCs, we believe that it is possible to create a registry suitable for nearly 100% of the Korean population with a handful of iPSC lines. There are few exceptions where autologous transfusions are needed, such as in the case of patients with rare blood types or alloimmunized patients receiving multiple transfusions, hence separate registry including these rare patients are required.

In this study, PB-MNCs from two patients with rare blood types (Jr(a-) and D–) were successfully used to generate iPSCs and differentiated into RBCs. The detailed protocol described in this research encompasses the entire process in a detailed manner, from the expansion of PB-MNCs and generation of iPSCs by transfection of episomal vectors, maintenance of iPSCs, and differentiation into erythroid lineage cells. Furthermore, this process makes use of our serum-free, xeno-free protocol which is compatible with the Good Manufacturing Practice (GMP). The use of autologous iPSC-derived RBCs from patients with rare blood types has been described in theory; however, to the best of our knowledge, this is the first report of banking rare blood type iPSCs for producing GMP-grade erythrocytes.

The major limitations for the clinical application of iPSC-derived RBCs are the inefficient RBC enucleation, difficulty of switching to adult-type globin, and the substantial number of RBCs (10^12^) needed to generate 1 unit of transfusable RBCs. Our protocol suffered from similar limitations, therefore the use of other small molecules and cytokines should be explored to overcome these hurdles [22–24]. Prior to using iPSC-derived RBCs clinically, further investigations are needed including: evaluation of RBC antigen profile, additional evaluation of iPSCs for genomic mutations, testing of the RBCs in agglutination assays, and/or safety testing of the RBCs in animal models. Lastly, in this study each cell line was differentiated into erythroid cells only once. Replicate experiments must be performed to make this protocol more robust and eliminate any variabilities.

Recently published articles deal with similar limitations and tries to find novel methods using modified cytokine mixtures and microenvironments. To produce GMP grade transfusable RBCs, many researchers are removing animal or human derived substances from prior protocols [53, 54]. Although it may be more costly and inefficient so far, xeno- and feeder-free methods are necessary to reduce possible side effects of manufactured RBCs when transfused to patients. Use of bioreactors, modifying microenvironments using macrophages and small molecules, and utilizing genetic alterations are currently being developed to enhance survival of mature RBCs, increase enucleation rate, and promote hemoglobin switching  [54–57].

## Conclusions

In conclusion, our findings demonstrate the feasibility of building a registry of iPSC clones from O D-positive donors and patients with rare blood types using a carefully developed serum-free, xeno-free protocol. The development of banked iPSCs for both research and clinical applications is an important step in the advancement of personalized medicine, and in fulfilling the need to establish specialized stem cell registry dedicated to RBC transfusions.

## Data Availability

The datasets used and analyzed during the current study are available from the corresponding author on reasonable request.

## Abbreviations

BMP4: Bone morphogenic protein 4
DD: Differentiation day
EB: Embryoid bodies
FGFa: Acidic fibroblast growth factor
GMP: Good Manufactory Practice
HPCs: Hematopoietic precursor cells
HSC: Hematopoietic stem cells
iPSC: Immortalized induced pluripotent stem cell
NANOG: NANOG homeobox
OCT4: Octamer-binding transcription factor 4
PB-MNCs: Peripheral blood mononuclear cells
PT-D: Post-transfection day
qRT-PCR: Quantitative real-time RT-PCR
RBC: Red blood cells
SCF: Stem cell factor
SSEA4: Stage-specific embryonic antigen 4
SOX2: Sex-determining region Y-box 2
VEGF: Vascular endothelial growth factor
